# Sulfonated Polystyrene Nanoparticles as Oleic Acid Diethanolamide Surfactant Nanocarriers for Enhanced Oil Recovery Processes

**DOI:** 10.3390/polym11091513

**Published:** 2019-09-17

**Authors:** Shalimar P. C. Caplan, Thaís B. G. Silva, Agatha D. S. Franscisco, Elizabeth R. Lachter, Regina S. V. Nascimento

**Affiliations:** Instituto de Química-Universidade Federal do Rio de Janeiro, Cidade Universitária, Rio de Janeiro, RJ 21941-909, Brasil; thaisbarros.gomes@gmail.com (T.B.G.S.); agathadensy@hotmail.com (A.D.S.F.); elizabeth.lachter@gmail.com (E.R.L.); rsandra@iq.ufrj.br (R.S.V.N.)

**Keywords:** surfactant release, polystyrene, nanocarriers, interfacial tension, enhanced oil recovery (EOR)

## Abstract

The aim of this study is the evaluation of partially sulfonated polystyrene nanoparticles (SPSNP) efficiency as nanocarriers for a non-ionic surfactant, oleic acid diethanolamide (OADA), in the reduction of the surfactant losses and the increase of oil recovery. The synthesized oleic acid diethanolamide was characterized by FTIR, ^1^H NMR, ^13^C NMR, surface tension (γ = 36.6 mN·m^−1^, CMC = 3.13 × 10^−4^ M) and interfacial tension of mineral oil/OADA aqueous solutions (IFT_eq_ = 0.07 mN·m^−1^). The nanoparticles (SPSNP) were obtained by emulsion polymerization of styrene, DVB and sodium 4-styrenesulfonate (St-S) in the presence of OADA aqueous solution and were characterized by FTIR and PCS. The results show that the presence of ionic groups in the polymer structure promoted a better nanoparticles suspensions′ stability, smaller particles production and more pronounced IFT reduction. The SPSNP obtained with an OADA concentration twenty times its CMC and 0.012 mol % of St-S presented a particle size around 66 nm and can act as efficient nanocarriers decreasing the water/oil interfacial tension to low values (0.07 mN·m^−1^) along the time, when in contact with the oil. Transport and oil recovery tests of the nanocarriers systems in an unconsolidated sand porous medium test show that the SPSNP do inhibit surfactant adsorption onto sand particles surface and induced an increase of oil recovery of up to about 13% relative to the water flooding oil recovery, probably due to a synergistic effect between the nanoparticles and surfactant action at the water/oil interface.

## 1. Introduction

Enhanced oil recovery (EOR) techniques have been employed to decrease the water to oil ratio in the exploiting of mature fields in order to increase the economic viability of those operations. It is expected that an EOR process will reduce the amount of residual oil not recovered by primary and secondary recovery, which can correspond to 35–50% of the original oil in place [1,2]. Several types of EOR processes have been employed, and surfactant injections are among the most successful ones. It reduces the water/oil interfacial tension and consequently lowers the capillary pressure, increasing the aqueous medium capacity to remove residual oil from the reservoirs’ pores. However, there is a great issue due to surfactant adsorption on the reservoirs rocks surface, causing a relevant loss of it which harms the process efficiency. To achieve a desirable oil recovery, it may be necessary to inject larger amounts of surfactant, but its high prices turn this process economically unfeasible [3,4].

To overcome this disadvantage, systems have been developed in order to stabilize surfactant molecules [5,6,7,8,9,10,11,12,13,14,15,16,17]. For example, Pei et al showed that O/W emulsion injection containing nanoparticles and surfactant can increase the tertiary oil recovery [7]. Similarly, a synergistic effect was observed of nanoparticles and surfactants in O/W emulsions stability [15] and a more intensified interfacial tension (IFT) reduction when silica nanoparticles were added into SDS solutions [16]. Other studies determined more specifically that particles can immobilize surfactants to act as surfactant carriers able to release at the target residual oil areas within the reservoir such as surfactant/beta-CD inclusion complexes inhibiting surfactant adsorption onto porous media [5]. SDS/silica particles reduced IFT probably due to surfactant release from the particles or to the effect of surfactant-coated particles [8,10] and carbonaceous nanoparticles MWNTs and CBs were used as surfactants carriers improving the tertiary oil recovery at shorter time intervals than a surfactant-only formulation [11]. Ehsan Nourafkan et al. [12] employed transport experiments through crushed sandstone grains showing that the surfactant adsorption was significantly lowered with the use of this strategy. The surfactant adsorption reduction was also evaluated using nanodroplets as surfactant nanocarriers. Microemulsions were obtained with hexane/brine systems with SDS and SPAM 80 as surfactants and were employed in core-flooding experiments. The results did show a reduction of surfactant adsorption and an increase in oil recovery efficiency [13].

Our research group has been studying the strategy of developing surfactant nanocarriers that can encapsulate/immobilize the surface active agent in a specific system and permeate through the reservoirs′ pores, delivering the surfactant molecules exclusively at the water/oil interface and consequently lowering the IFT [6,14,17].

However, for this strategy to work in oil fields, it would be necessary to employ surfactants able to promote a great lowering of the water/oil interfacial tension values. Most of the studies from the literature [8,11,15,16] that evaluate the performance of surfactant carriers in EOR experiments employ commercial surfactants, such as SDS, which are not able to produce that degree of interfacial tension reduction. For this reason, it was decided to use oleic acid diethanolamide (OADA) as a nonionic surfactant in the present work, since previous results from our group have shown its capacity to lower the water/oil IFT to values inferior to 1.0 mN/m [17].

Polystyrene nanoparticles (PSNP) are gaining interest for application in many different fields, such as reinforcing fillers for polymeric nanocomposites and polymeric films for dielectric utilization [18]. Besides, due to their submicron sizes, they can act as a polymeric delivery system, i.e., drug carriers [19]. One of the most common techniques used for the production of these nanoparticles is the emulsion polymerization reaction, but the size control and suspension stability of the obtained nanoparticles is sometimes difficult to achieve [20]. In this mechanism, the monomer is emulsified with the aid of a surfactant, since it is insoluble in the aqueous phase. For that, the surfactant concentration must be above the critical micelle concentration (CMC). On the other hand, the initiator is water soluble and consequently, the polymerization will start with the few monomer molecules dissolved in the continuous phase. Subsequently, the first oligoradicals formed can be absorbed by micelles containing the monomers, and that gives a continuation to the polymerization process. This mechanism proceeds until monomer/radical depletion occurs, producing 50–300 nm diameter particles [21,22,23,24,25,26]. In addition to these factors, Wutzel and Samhaber′s study [27] has investigated the influence of higher amounts of initiator and/or surfactants on the final particle size, since the number of particles in the first stage of the reaction is related to the final particle diameter (d ∝ N^−1/3^). Accordingly, experimental conditions, such as emulsifier type and concentration, initiator, monomer chemical structure, stirring conditions and temperature have an influence on the size and other particles’ properties.

Particle size is an essential parameter in order to ensure that the carrier will be able to permeate through the pores of the reservoir, as would be the stability of the particles aqueous suspensions, since agglomerates could clog the pore throats. To obtain particles smaller than 100 nm and also to prevent nanoparticle agglomeration, sulfonated styrene was employed as a monomer in the present work to obtain partially sulfonated polystyrene nanoparticles (SPSNP). The presence of ionic groups in the polymer structure should increase the nanoparticles suspensions′ stability. Their performance as OADA carriers was compared with the one obtained with non-ionic polystyrene nanoparticles (NPPS). 

Therefore, the main objective of this work was to develop PSNP and SPSNP that would be able to carry oleic acid diethanolamide molecules through reservoirs porous medium and deliver it at the water/oil interface, reducing interfacial tension and increasing oil production.

## 2. Materials and Methods 

### 2.1. Materials

Styrene (St), sodium 4-styrene-sulfonate (St-S), potassium peroxydisulfate (KPS), diethanolamine and n-heptane were purchased from Sigma-Aldrich Brasil Ltda. (São Paulo, Brazil). NaCl, n-hexane and K_2_CO_3_ were purchased from Vetec Química Fina Ltda. (Rio de Janeiro, Brazil). Mineral oil (viscosity: 21.09 mPa·S and density: 0.86 g/cm³, both at 25 °C) was obtained from B.Herzog (Rio de Janeiro, Brazil). All reagents were used without any previous treatment. Distilled and deionized water was used throughout the work. Sand used as the porous medium was a donation from Mineração Jundu Ltda. (Descalvado, Brazil). Crude oil was a donation from Petrobras (Rio de Janeiro, Brasil).

### 2.2. Experimental Procedures

#### 2.2.1. Oleic Acid Diethanolamide (OADA) Synthesis and Characterization

**Figure 1:**
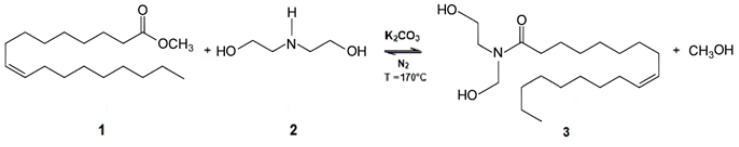
OADA synthesis scheme where the main chemical species involved were highlighted as **1**: Methyl oleate, **2**: diethanolamine and **3**: OADA.

The synthesis of oleic acid diethanolamide (OADA) was performed by the esterification reaction of methyl oleate (1) with diethanolamine (2) in a 1:3 ester:amine molar ratio, as shown in Figure 1 and described in the literature [17,28]. Under nitrogen atmosphere, the system was heated until it reached 170 °C and then, 5 (*w*/*w*) % of catalyst (K_2_CO_3_) were added. After that, it was refluxed for 8 h at 170 °C under constant magnetic stirring. 

To eliminate unreacted species, the mixture was purified using solvent extraction (saturated NaCl solution and hexane). The obtained product was characterized through FTIR, ^1^H NMR and ^13^C NMR.

#### 2.2.2. Synthesis of Crosslinked Polystyrene Nanoparticles (NPPS)

Based on the systems studied by Avila et al. [6], styrene (10 mL), a specific amount of DVB and an OADA aqueous solution were added to a three-necked flask containing 250 mL of deionized water under nitrogen flow, equipped with a condenser and under magnetic stirring. Here, OADA was used both as the emulsifier for the emulsion polymerization and as the surfactant to be carried. Due to that, the OADA content used was based on an excess of its critical micelle concentration, which was determined by the surface tension method [29,30]. After homogenization of the system, 50 mg of KPS were added as initiator. Then, the mixture was stirred for 6 h at 80 °C. The obtained emulsion was purified by separation and enrichment based on centrifugal ultrafiltration (filter membrane Amicon Ultra NMWCO 20K, Merck Millipore Brasil, Barueri, Brazil). After each centrifugation cycle, deionized water was added, allowing the nanoparticles to be washed three times to eliminate unreacted species and free surfactant that remained in the aqueous media.

#### 2.2.3. Synthesis of Crosslinked Sulfonated Polystyrene Nanoparticles (SPSNP)

A certain amount of styrene (St) was partially substituted by sodium 4-styrenesulfonate (St-S) under the same conditions, and the proportions between total monomer, initiator and surfactant amounts were maintained [31]. The main formulations for the nanoparticles polymerization reactions studied in this work are listed in Table 1. This was accomplished by keeping constant the total monomer amount (87.2 mmol), while the mol % for the two monomers was altered. The crosslinking agent employed was DVB, in 0.3 mL for all formulations. The obtained emulsion was purified by separation and enrichment as described in the previous section.

#### 2.2.4. Nanoparticles Characterization

PSNP and SPSNP were characterized by infrared spectrometry (FTIR) in the 400–4000 cm^−1^ range, using a Fourier transform infrared spectrometer (Nicolet 740 FTIR from Thermo Fisher Scientific, Waltham, Massachusetts, EUA) with a DTGS KBr detector and a beam splitter. Conventional preparation techniques of KBr pellets for solids were employed. Particles number-average diameter and polydispersity index (PdI) results were obtained by photon correlation spectroscopy (PCS, Malvern Zetasizer–MAL 1013334 from Malvern Panalytical Brasil, São Paulo, Brasil).

The nanoparticles stability during storage and agglomeration tendency in deionized water at room temperature was investigated by monitoring particle size (hydrodynamic diameter obtained by PCS) for 60 days.

#### 2.2.5. Superficial and Interfacial Tension Measurements

The surface tension of the nanoparticles/water systems was measured using the Wilhelm plate method, and the interfacial tension (IFT) between suspensions with different nanoparticles concentrations and mineral oil, through the Du Nouy Method at constant temperature (28 ± 0.5 °C). For all these measurements, the apparatus used was a Krüss K100 tensiometer (Hamburg, Germany). Also, through IFT reduction with time, it was investigated the occurrence of the surfactant (OADA) release at the water/oil interface. For low IFT systems, like the ones obtained by surfactant solutions in water and some SPSNP suspensions in water, the Krüss SITE 100 spinning drop tensiometer was used at 28 °C. The rotating tube of the system was filled with these prepared solutions/suspensions, and, during the analysis, about 3 µL of mineral oil were added. Then, the tension was measured along the time, until it reached equilibrium. All measurements were performed in triplicate.

#### 2.2.6. Quantification of Surfactant Retention by NPPS

To quantify the amount of OADA that remained on the nanoparticles′ structure after the synthesis process, it was measured the total organic carbon in the supernatant after each centrifugation cycle by a Total Organic Carbon (TOC) Analyzer (TOC-L from Shimadzu Brasil, Barueri, Brazil), considering that the carbon present was from the free surfactant. As a result, the difference between the OADA amount added before the reaction and the one quantified by TOC analysis was considered to be the one that corresponds to the OADA immobilized or encapsulated in the nanoparticles, according to Equation (1) [32].

*Q*_e_ = (*C*_i_ − *C*_e_)/*m* × *V* (1)

*C*_i_ and *C*_e_ are the initial and equilibrium (in the supernatant phase) concentrations (mg/L), *m* is the nanoparticle mass (g) and *V* the solution volume (250 mL).

The retained surfactant percentage (Retention%) was calculated from Equation (2).

(Retention%) = (*C*_i_ − *C*_e_)/*C*_e_ × 100 (2)

#### 2.2.7. Sand Pre-Treatment and Characterization

The sand was washed with a 0.1% HCl solution and later thoroughly washed with deionized water to remove any soluble impurities. To remove any organic compounds still present, it was calcined at 600 °C for 12 h. The resulting sand presented 30% of porosity and size distribution between 362 and 635 µm.

The mineral composition of the sand used as porous media was determined by X-ray diffraction (XRD) using a RIGAKU Ultimate IV X-ray diffractometer (Rigaku Corporation, Tokyo, Japan) which recorded the 2θ range 10–80° at a scan rate of 0.02°/min, using CuKα (λ = 1.54 Å) radiation. Also, thermogravimetric analysis (thermogravimetric analyzer TGA-51 from Shimadzu Brasil, Barueri, Brazil) was carried out in a heating rate of 10 °C/min and temperature range of 25–800 °C under a O_2_ atmosphere in order to evaluate the existence of organic matter adsorbed. 

#### 2.2.8. Transport Test Evaluation 

To ensure that the prepared nanoparticles suspensions could be transported through a porous medium, their transport and adsorption behavior were analyzed by experiments in an unconsolidated sand porous medium column test. The porous medium was constructed in a liquid chromatography column (diameter 2.5 cm, length 15 cm), filled with sand and sealed with two PTFE (polytetrafluoroethylene) end fittings, connected to a peristaltic pump (Masterflex^®^ L/S peristaltic pumps, Cole-Parmer, Vernon Hills, Illinois, USA). The test consists of subsequent fluid injections in the column: 1 pore volume (PV) of distilled water, 3 PV of surfactant solution or nanoparticle suspension and finally 3 PV of distilled water. The concentration of the active substance (surfactant or nanoparticle) in the injected fluid was 0.1 (*m*/*v*) %, and the pumping flow rate was 0.1 mL/min. During the experiment, every ten ml of effluent were collected and analyzed by TOC detection for the surfactant-only injections or by UV–Vis spectrophotometry at 400 nm in the case of nanoparticles injection. The active substance content in the effluents allowed to plot the breakthrough curves, i.e., the relative concentration *C*/*C*_0_ (*C*—concentration in the effluent and *C*_0_—initial concentration injected) as a function of the pore volume [11,33]. Besides, to evaluate the content of adsorbed material in the medium, a graph of cumulative content recovered versus the pore volumes injected was obtained.

#### 2.2.9. Oil Recovery Tests

**Figure 2:**
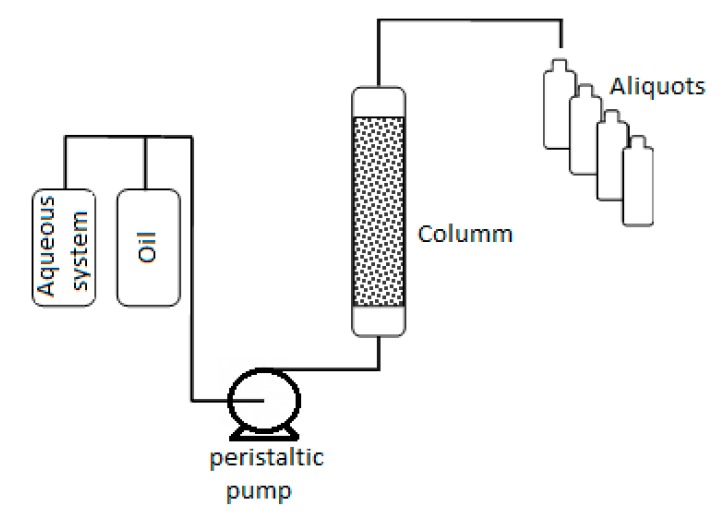
Oil displacement set-up scheme.

These tests were conducted using the same system described in the previous section to evaluate the efficiency of the surfactant delivery system employed in recovering the oil, as shown in Figure 2. Initially, 1 PVs of a mixture of 50% crude oil, 25% mineral oil and 25% heptane, here used to simulate a paraffinic oil, was added and then, a sand presenting the same characteristics as the one used in the previous experiment was used to fill the column. Subsequently, 3 PVs of deionized water were injected at 1 mL/min as displacing fluid and afterwards 1 PV of the aqueous nanoparticles′ suspensions, which corresponds to tertiary oil recovery. At the end, 2 PVs of deionized water were injected. The aqueous systems used were a surfactant solution (0.006% OADA aqueous solution) or 0.1 wt % SPSNP solutions with nanoparticles of different sulfonate contents.

Effluents were collected in 10 mL graduated cylinders and, based on the oil volume measured from every effluent collected, materials balance calculations were carried out to evaluate the oil recovery as a function of fluid injected [11,14,34].

## 3. Results and Discussion

### 3.1. OADA Characterization

**Figure 3:**
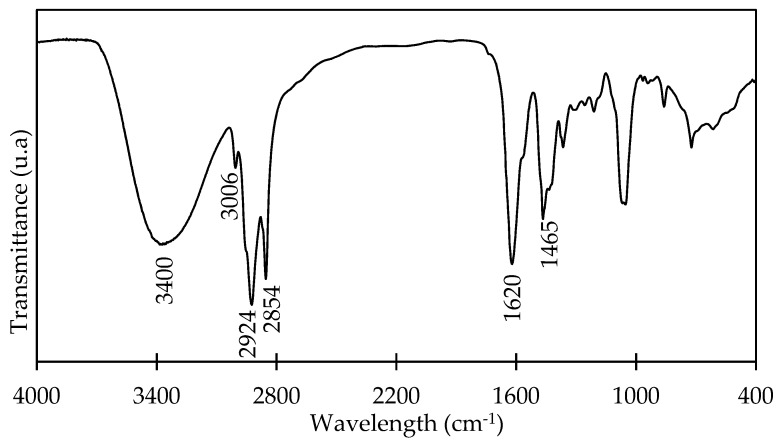
FTIR spectrum of OADA.

The FTIR spectrum for OADA is shown in Figure 3. A broad-band at 3400 cm^−1^ was attributed to stretching modes of OH group of alcohol and a band at 1065 cm^−1^, for stretching modes of C–O bond of primary alcohols. The stretching vibrations of amide carbonyl were observed at 1620 cm^−1^ and C–N stretching at 1465 cm^−1^. Asymmetrical and symmetrical stretchings of –CH_2_ and –CH_3_ of long chain fatty acid appeared at 2924 and 2854 cm^−1^, respectively. The disappearance of –NH– angular vibration at 1550 cm^−1^ confirmed an efficient elimination of amine species that were used in excess in the reaction.

**Figure 4:**
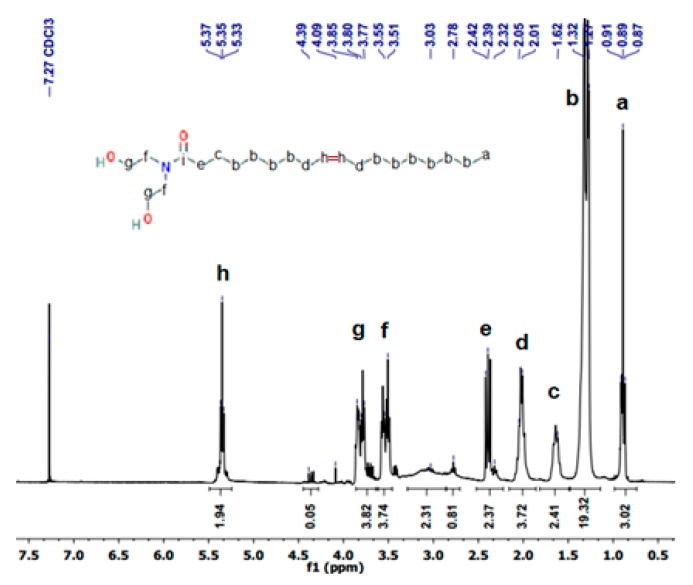
^1^H NMR spectrum of OADA.

The ^1^H NMR spectra of OADA is displayed in Figure 4, where some characteristic peaks were identified: Terminal proton (a) of –CH_3_ at δ = 0.89 ppm, –CH_2_ proton (b) present in the long fatty chain at δ = 1.27 ppm, –CH_2_ proton (e) adjacent to amide –C=O observed at δ = 2.4 ppm, proton (g) of –CH_2_ near hydroxyl appeared at δ = 3.9 ppm, proton (h) of –C=C– of unsaturated fatty acid chain at about δ = 5.35 ppm and the peak at about δ = 3.53 ppm is assigned to -CH_2_ adjacent to amide nitrogen. These peaks were also confirmed in the literature [28,35].

**Figure 5:**
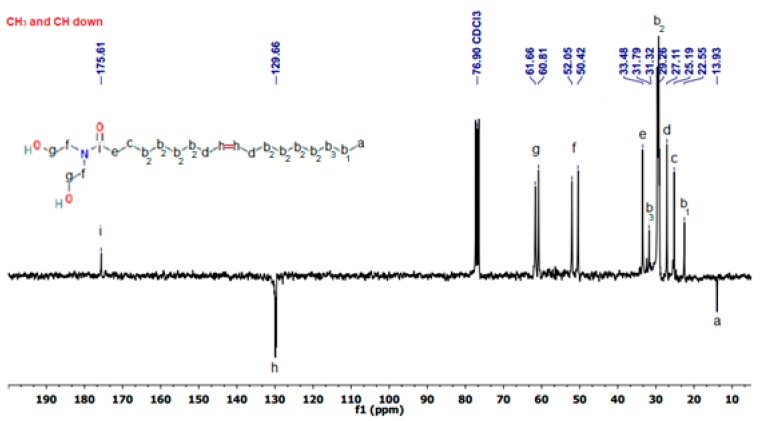
^13^C NMR-APT spectrum of OADA.

Figure 5 shows the ^13^C NMR spectrum of OADA, using attached proton test (APT) technique, and the structure obtained by ^1^H NMR analysis was confirmed by the results from ^13^C NMR. The characteristic peak of –CH_3_, present in the ester methyl group (methyl oleate), which was used as a reagent, was not present, and that confirms the completion of the reaction.

**Figure 6:**
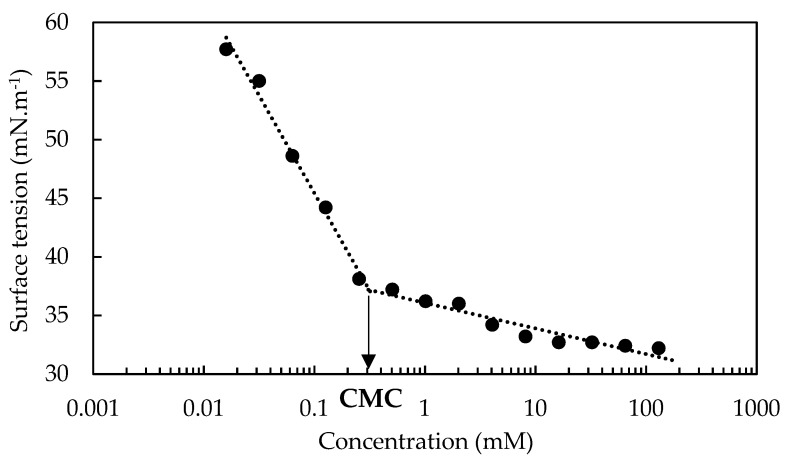
Surface tension as a function of the logarithm of surfactant concentrations for the CMC determination.

Since OADA was used in this work as a surfactant for both polystyrene polymerization and enhanced oil recovery processes, it was necessary to determine its critical micelle concentration. For that, surface tension measurements were performed, and a graph of surface tension versus OADA concentration was plotted, as shown in Figure 6. The critical micelle concentration corresponds to the inflection of the curve in the graph. The oleic acid diethanolamide produced a relevant reduction of the water surface tension (72.3 mN·m^−1^), reaching values less than 40 mN·m^−1^, which is related to a superior wetting agents category [30,36]. As expected, the surface tension decreased as concentration increased, and it was observed a clear breakpoint related to this condition of critical micelle concentration (CMC): γ_CMC_ = 36.6 mN/m and CMC = 3.13 × 10^−4^ M or 115.3 mg/L. Greater concentration values caused a lower reduction of surface tension.

**Figure 7:**
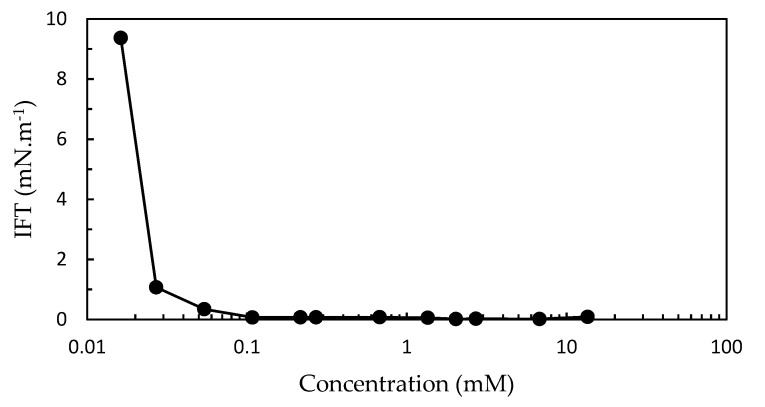
IFT as a function of surfactant concentration.

The important effect of the OADA concentration on the water/mineral oil interfacial tension (IFT) is shown in Figure 7.

It was observed that at very low OADA concentrations occurred a drastic IFT reduction when compared to water/mineral oil interfacial tension (12.6 mN·m^−1^), which is in accordance with other surfactants previously studied [37]. At a concentration of 0.10 mM (IFT = 0.070 mN·m^−1^), the curve seems to have reached an equilibrium plateau, although greater concentrations still caused a discrete IFT decrease until reaching a minimum at 0.017 mN·m^−1^ for the solution containing 2.0 mM of OADA.

### 3.2. Nanoparticles Characterization

**Figure 8:**
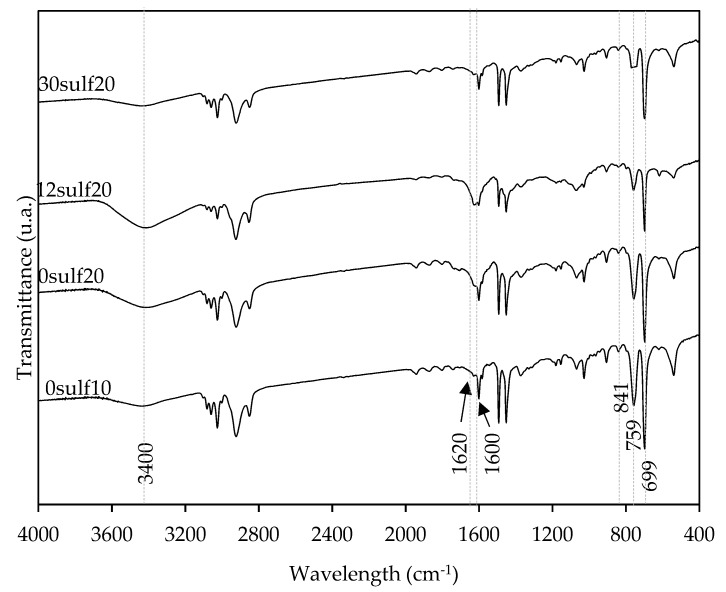
Polystyrene nanoparticles FTIR spectra with different OADA and sulfonate contents: 30sulf20, 12sulf20, 0sulf10 and 0sulf20, whose polymerization formulations are described in Table 1.

FTIR and PCS analyses were performed to investigate the presence of surfactant and sulfonate functional groups in the synthesized nanoparticles polymer structure, and their influence on the nanoparticles′ particle size. Figure 8 shows the FTIR spectra for: 0sulf10, 0sulf20, 12sulf20 and 30sulf20 nanoparticles.

Comparing the spectra for the nanoparticles with different amounts of OADA, it was noted that the bands referring to C–H stretching vibration (CH_3_, CH_2_ groups) at 2854 and 2924 cm^−1^, C=O stretching vibration of tertiary amide at 1620 cm^−1^ and C–N stretching vibrations at 1465 cm^−1^ were present for all samples. However, it was possible to perceive some differences related to the amount of retained surfactant in the 0sulf20 and 12sulf20 spectra. It was observed that in the samples′ spectra, especially for 12sulf20, there are relatively greater intensities of bands at 1620 and 3400 cm^−1^, which can be attributed to a larger OADA amount present in the nanoparticles.

Additionally, the FTIR spectra are similar except for some bands, such as the one at 699 cm^−1^ which is characteristic of the out-of-plane skeleton bending vibrations of benzene ring. Another is at 759 cm^−1^ which refers to the out-of-plane bending vibration of the five –CH– groups in the ring of the monosubstituted benzene ring. It was noticed that the intensity of these bands is lower when increasing the sulfonation degree, as was also observed by Yang et al [38]. It is also reported that the intensity of the band at 841 cm^−1^, referring to a para substitution of the ring, decreased with SO_3_ substitution [38,39].

### 3.3. Effect of Crosslinking Agent and Surfactantconcentration on the Non-Sulfonated Polysturene Nanoparticles (NPPS) Size and on the Oil/Water Interfacial Tension

Some studies have shown that there is not a clear tendency of increasing or decreasing particle size obtained by polymerization reactions carried out with increasing of crosslinking agent content [31,39]. Despite that, it could be expected that an increase of DVB amount could favor the production of polystyrene particles presenting smaller diameter and pore sizes. Based on the fact that small particle sizes with surfactant controlled release ability are preferred for EOR application, the aim of this study was the determination of the DVB amount that better satisfies this condition by means of monitoring particle size and IFT measurements.

Also, the emulsion polymerization reactions were performed using an OADA concentration above the critical micelle concentration (CMC) in order to obtain the PSNP in appropriate sizes since Avila et al. [6] noted that the PS nanoparticle sizes tended to decrease when it was used greater amounts of surfactant. So, in this work, a similar methodology was used, testing not only the influence of a more non-polar non-ionic surfactant, as OADA, but also the effect of greater or equal surfactant concentrations than the ones investigated in that work. Table 2 summarizes the particle size results obtained by PCS and the interfacial tension values for water/mineral oil systems measured by the Du Nouy ring method, after 24 h.

As previously reported [6,39], there is an expected trend of particle size decrease with the increase of crosslink agent concentrations. Nevertheless, the reduction of nanoparticles size obtained with 4.5–9.0 (*v*/*v*) % of OADA was not significant. This might be due to the inter-crosslinking between the precursor nanoparticles [31]. The results obtained with samples 03 and 07 show that the greater amounts of surfactant favored the formation of smaller droplets containing monomer during the polymerization and led to the decrease of the nanoparticle size. To achieve a system suitable for enhanced oil recovery, the PSNP must present not only a nanometric size, but also a capacity to reduce IFT. Therefore, it was investigated which conditions lead to minimal IFT values and this was achieved by 03 and 07 samples whose PSNP resulted from 3 (*v*/*v*) % of DVB. The IFT reduction for the 07 sample was more pronounced probably due to a higher surfactant retention. Therefore, this condition of 3 (*v*/*v*) % of DVB was adopted for the subsequent experiments. The IFT reduction for the 07 sample was more pronounced probably due to a higher surfactant retention which is better discussed in Section 3.8.

### 3.4. Effect of the Sulfonate Group Content on SPSNP Size

**Figure 9:**
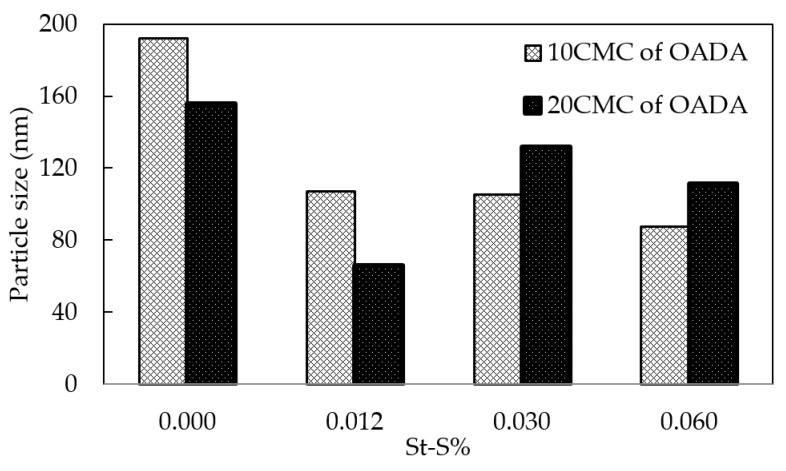
Influence of St-S content on SPSNP particle size prepared with different OADA concentrations: 10CMC and 20CMC.

Sulfonate groups are present in many known surfactants such as sodium dodecylbenzene sulfonate (SDBS) which have been applied to enhanced oil recovery [11,12,13]. Besides, some other studies [31,39,40,41] showed that these groups have an influence on the polymerization reaction and the obtained particle sizes. Particles obtained by polymerization carried out in the presence of the ionic sodium styrene sulfonate are more dispersible in water and more stable since the ionic charges on the particles′ surface prevent agglomeration. In the present study, polystyrene nanoparticles containing 0–0.06 mol % of sodium styrene sulfonate were analyzed by PCS and IFT measurements which were performed in a spinning drop tensiometer. It was observed an effect of the sulfonate groups content on the average diameter obtained by numeric data (PCS), as shown in Figure 9.

These results indicate that the comonomer containing sulfonate groups would be acting as a stabilizer and nucleation promoter for the particles. Due to the probability that these ionic charges would stay on the particles surface, the particles would repel each other and prevent the agglomeration of precursor particles. On the other hand, in the same way, that larger surfactant concentrations result in greater quantities of particles formed during polymerization, the larger amounts of water soluble comonomer (St-S) will favor particle nucleation. Therefore, as a consequence, the number of particles increases and the particle size decreases since the total monomer amount is the same in the samples analyzed [42,43,44].

Zhang [45] showed this same tendency of particle size decrease at very low St-S content, followed by a sudden particle size increase. This behavior may be explained by a lower concentration of monomer molecules present in the micellar core since greater fractions of comonomers (St-S) were solubilized in the aqueous phase. The change, however, is due to a variation in the nucleation mechanism. Initially, when monomer species are almost completely hydrophobic, the major nucleation mechanism occurs in the micellar core [46]. Further increase of St-S concentration shifts the nucleation (locus of polymerization) to the aqueous medium, occurring homogeneous particle nucleation which causes mainly the growth of oligomers, that would be already stabilized by the emulsifier. Accordingly, the formed particles present greater sizes than the ones from micelles stabilized by the emulsifier [47]. Therefore, it was observed a minimal size of 66 nm for the sample produced with 0.012% of St-S and ten times the CMC of OADA.

Due to the higher total particle surface area, which could be being stabilized by the sulfonated groups, the final particle size distribution was narrower and the polydispersity index was lower than 0.1, indicating that the nanoparticles were relatively monodisperse.

### 3.5. Effect of the Sulfonate Group Content on Water/Oil IFT Values for the Systems Containing SPSNP

**Figure 10:**
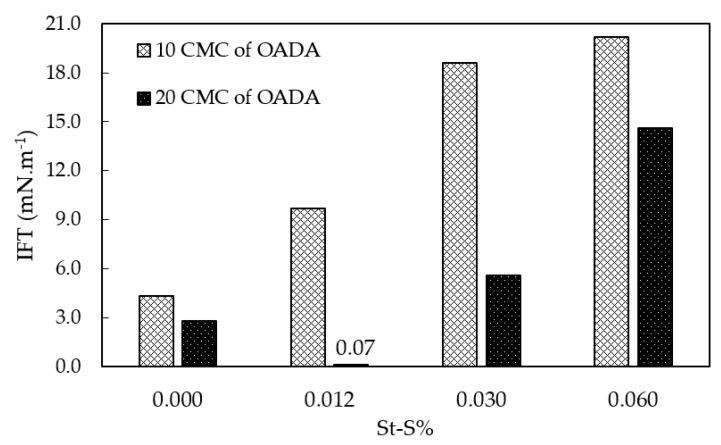
Influence of St-S content on the interfacial tension between 0.1 (*m*/*v*) % SPSNP suspensions in deionized water and mineral oil.

Figure 10 shows the IFT results as function of the sulfonate group and OADA contents. This graph presents the IFT values which correspond to the final value in the experiment (Spinning Drop measurements) when the IFT versus time curve reached an equilibrium plateau. SPSNP synthesized in the presence of a greater amount of surfactant (20CMC of OADA) have shown to be more effective in the reduction of water/oil interfacial tension when mineral oil was used as the oil phase. This tendency can be explained by a larger surfactant content retained in the nanoparticles′ structure, as indicated by the 03 and 07 samples analysis as described in Table 2. Further investigations on retained surfactant quantities are described in Section 3.8.

In addition, taking into consideration that the substitution of styrene monomers by sulfonated styrene causes a reduction in particle size (as shown in Figure 9), it could be expected a higher interfacial activity for these systems, since they show greater area/volume ratio. Low concentrations of St-S favor the reduction of interfacial tension, because smaller nanoparticles are more effective in displacing other molecules at the interface [16]. However, at higher sulfonate group concentrations, this tendency is not observed. The nanoparticles become more soluble in the aqueous medium to such an extent that they do not migrate to the water/oil interface any longer. Therefore, the number of active substances (nanoparticles + surfactant system or free nanoparticles or free surfactant molecules) at the water/oil interface is reduced, increasing the interfacial tension. Considering a balance of these factors, it was determined which system would be derived from the optimized conditions. The system that showed minimal particle size and interfacial tension in the range of ultra-low IFT was the one synthesized with 0.012 mol % St-S content and an OADA concentration of twenty times the CMC.

### 3.6. Surfactant Controlled Release Study

**Figure 11:**
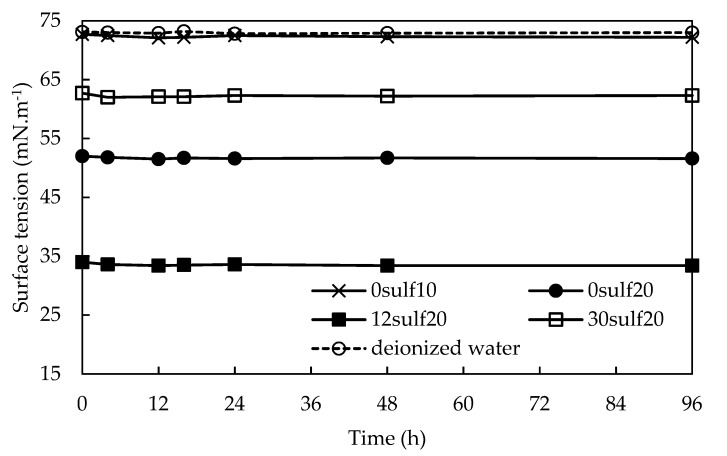
Surface tension behavior over the time for systems containing 0.1 (*m*/*v*) %. Measurements were performed using the Wilhem plate method (temperature: 25 ± 0.5 °C).

In order to better evaluate if the prepared systems could be applied as surfactant-controlled release nanocarriers, surface and interfacial tension measurements were performed to evaluate their dependence with time. For that, suspensions containing 0.1 (*m*/*v*) % of 0sulf10, 0sulf20, 12sulf20 and 30sulf20 nanoparticles were prepared and surface tension values were collected until they reached an equilibrium. Then, this property was monitored over the first 96 h, as revealed in Figure 11. As expected, the surface tension values for all samples did not present any significant variation, meaning that a premature surfactant loss in the aqueous phase during an EOR process would be negligible. This can be explained by the existence of hydrophobic interactions between the polystyrene chains and the oleic acid diethanolamide molecules, especially through their long fatty chain, that turns difficult their release in a polar medium. In other studies by our Group [17], it was achieved similar results using nonionic surfactants in mesoporous silica systems.

**Figure 12:**
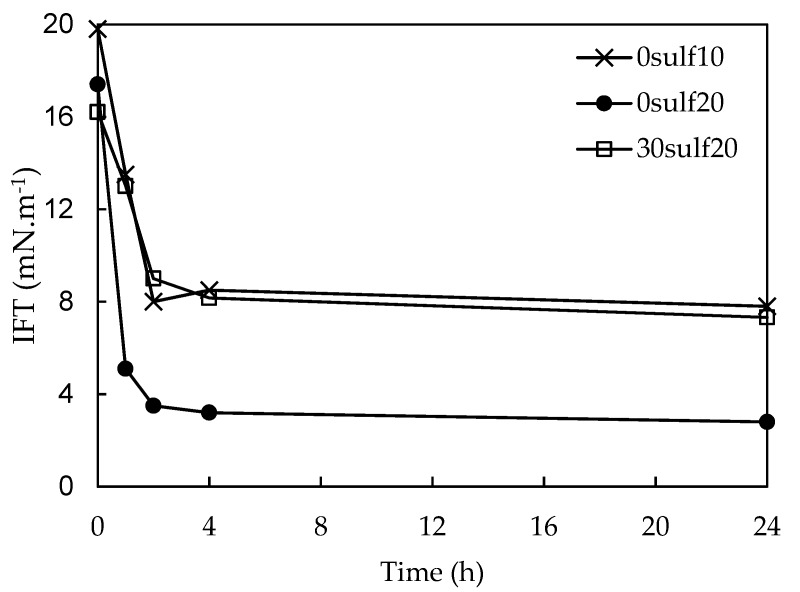
IFT behavior over time for systems containing 0.1 (*m*/*v*) % suspensions and mineral oil. Measurements were carried out using the Du Nouy method (temperature: 25 ± 0.5 °C).

**Figure 13:**
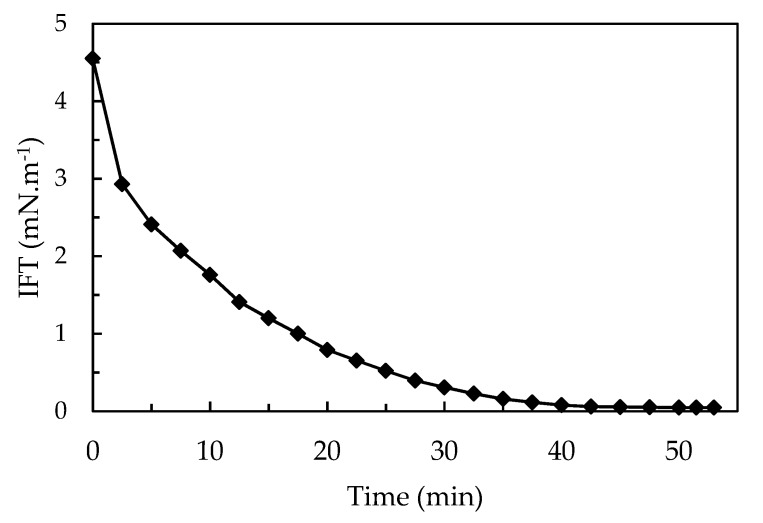
Interfacial tension (IFT) for the 12sulf20 SPSNP suspension (0.1 *m*/*v* %) and mineral oil as a function of time. Measurements were performed using Spinning Drop method.

Afterwards, these aqueous systems were placed in contact with oil to proceed with the interfacial tension (IFT) measurements. Figure 12 shows a typical L-shape where an IFT reduction occurs during the first two hours of analysis and, after that period, the variations were not relevant. Freitas et al. [17] and Rosestolato et al. [14] identified a similar behavior in which the IFT decreased gradually during the first hours probably due to the migration of the surfactant molecules away from the nanoparticles to the water/oil interface where they replace the solvent molecules until the IFT reached an equilibrium plateau. The system containing 12sulf20 nanoparticles readily reached IFT values lower than 1.0 mN·m^−1^ when it was measured by the Du Nouy method. Since the Du Nouy method is not able to measure values below 1.0 mN·m^−1^, the IFT measurements for the 12sulf20 system were carried out in the spinning drop tensiometer and the results are displayed in Figure 13.

The Figure 13 shows that the 12sulf20 resulted in lower IFT values. Also, it is possible to note that this system acts at the interface and reaches an equilibrium after a shorter time interval in comparison with the other systems.

Besides, it was prepared an OADA solution containing 0.006% of free surfactant that corresponds to a theoretical system where all surfactant used in the polymerization would have been retained and then liberated at the interface. The spinning drop measurements for the system formed by this solution in contact with the mineral oil show an equilibrium IFT of 9.3 mN·m^−1^, obtained after 3 h. Comparing its performance with the results for the nanoparticles systems, shown in Figure 12 and Figure 13, it is possible to suppose that a synergistic effect did occur in these systems, since the 12sulf20 nanoparticles presented a much reduced IFT values and shortened the time necessary to establish the equilibrium. Similar results were observed in the literature [9,10,17].

Therefore, the results have shown that the sulfonated polystyrene nanoparticles (12sulf20) can act as efficient nanocarriers, causing an interfacial tension decrease along the time when in contact with the oil, reaching very low IFT values in a short time. 

### 3.7. Nanoparticles Storage Ability

**Figure 14:**
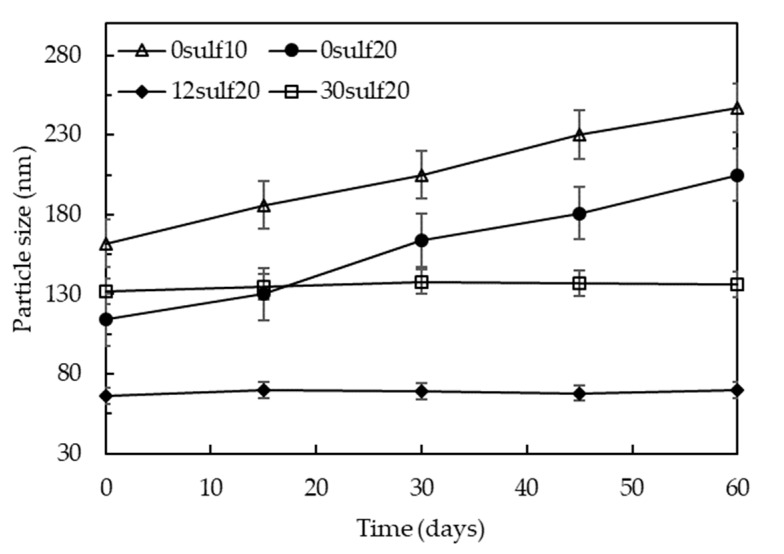
Influence of sulfonate groups on nanoparticles stabilization.

Figure 14 shows the nanoparticles stability in aqueous media over storage time, revealing that the particle size tends to increase along the time when sulfonate groups are absent in the nanoparticles′ structure. Considering that all nanoparticles analyzed in these experiments were washed with deionized water to remove free surfactant molecules, the elimination of OADA excess probably lead to the destabilization of the PSNP systems, causing agglomeration of the smaller nanoparticles. In contrast, the SPSNP, which present sulfonate groups derived from the St-S polymerization, maintained their particle size over all the storage time, even removing the OADA excess. The anionic groups distributed along the polymer chains cause electrostatic repulsion among the nanoparticles, inhibiting their agglomeration. Based on these results, sulfonated polystyrene proved to be more suitable to be applied to transport through reservoirs′ pores without causing any clogs by agglomerating.

### 3.8. Quantification of Surfactant Retention by Nanoparticles

Table 3 summarizes the experimental results obtained for the quantification of OADA retained in the nanoparticles and shows the effect of the sulfonate group content and the OADA concentration used in the polymerization medium on the retained surfactant percentage (Retention%) and on the surfactant content in the nanoparticles (*Q*_e_).

Surprisingly, the use of greater amounts of OADA in the polymerization process did not result in an increase on the retained surfactant amount (*Q*_e_) as could be expected when comparing with the results of the adsorption process as described in the literature [17,32,34]. Since OADA presents a double role in the nanoparticles′ formation, its consumption may occur during the formation of a greater number of micelles and nuclei, producing smaller nanoparticles, as observed in Figure 9, and consequently resting less OADA molecules available to be immobilized or encapsulated in the nanocarriers. However, the larger amounts of surfactant retained were obtained by the 0sulf20 and 12sulf20 samples. This result corroborates the observations conducted through FTIR and IFT analysis. Besides, it is concluded that the surfactant content in the nanoparticles is determinant to obtain a low IFT system. The small nanoparticle size (preferably below 100 nm) and the presence of sulfonate groups are also relevant factors to produce an effective system.

The results derived from the measurement of the surfactant retention capacity allowed to observe another relevant aspect that is related to the type of nucleation mechanism. Higher St-S amounts in the polymerization medium favor homogeneous particle nucleation [40], since anion-charged oligomers tend to be more soluble in water, resulting in more OADA molecules free in the medium, which are removed in the purification step. This would explain why the retained OADA in the 30sulf20 SPSNP was the lowest observed.

### 3.9. Sand Characterization

The adsorption process that occurs during the EOR surfactant flooding depends on the type of surfactant used and on the morphological characteristics and mineralogical composition of the rock [48,49]. The surface charge on the porous medium must be considered to understand the adsorption mechanism involved in the transport test [50,51,52]. It is known that silica normally adsorbs cationic surfactants, because, at neutral pH, silica surface presents a weak negative charge, affecting the efficiency of a surfactant flooding [53]. Considering these factors, a semi-quantitative phase analysis of the XRD spectrum was carried out to determine the mineralogical composition of the sand. For that, the diffraction pattern was analyzed with the software *Match!* (Crystal Impact GbR, Bonn, Germany) using IUCr/COD/AMCSD as the reference database. The composition of the sand was 71.9% of quartz and 28.1% of berlinite (AlPO_4_). Berlinite is isostructural with α-quartz and presents a negatively charged surface at neutral pH [54,55,56].

TGA measurements did not identified any organic matter or other volatile compound loss during the sample heating which ensures that the preliminary sand treatment was efficient.

### 3.10. Transport Test Evaluation

**Figure 15:**
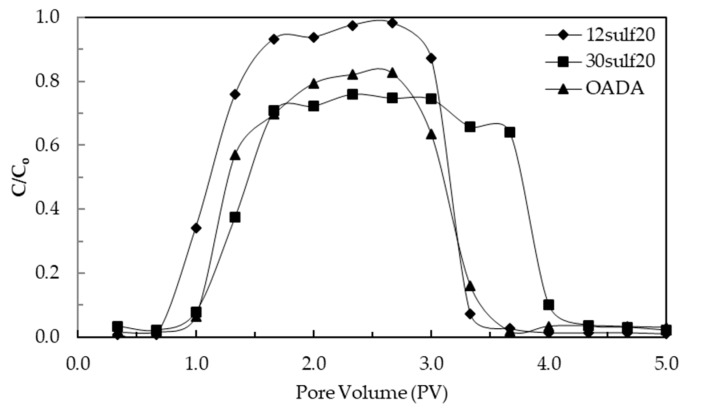
Breakthrough curves for suspensions of 12sulf20 SPSNP and 30sulf20 SPSNP and OADA solution (0.2 *m*%/*v*%).

Transport tests were performed to evaluate the surfactant and nanoparticles losses that may occur during transport, as can be seen by the results in Figure 15. Also, nanoparticles may cause pores clogging depending on their size and interactions with the porous media. The hydroxyl groups present in the OADA structure and sulfonate groups present in the nanoparticles could interact with silanol groups of silica, which represents the main component of sandstone reservoirs, and, therefore, the adsorption of these materials would be favored. Some studies have already reported that anionic surfactants containing sulfonates as head groups can adsorb on positively charged clay edges contained in sandstones [3,57,58,59]. In a less extent, nonionic surfactants, that contain polar groups, can adsorb onto sand surface due to hydrogen bonds and weak hydrophobic interactions between them, causing surfactant loss during transport through the medium [60,61,62,63]. For that reason, this experiment must be conducted before the oil displacement test, when several conditions may be applied.

The analysis of the Breakthrough curves (Figure 15) shows that the systems have different elution profiles. The free surfactant (OADA) concentration at the plateau did not reach the initial concentration of the injected system, which means that a fraction of it was retained on the porous sand surface. In the nanoparticles’ fluids curves, it is noted that the lower the sulfonate group content in the nanoparticles, greater was its percolation capacity in the porous medium. In the same way as it was observed for the curve related to the OADA solution, the concentrations of the 30sulf20 nanoparticle suspension at the plateau was inferior to the injected concentration and also showed a delay in its elution from the porous medium, similar to a tail [64], probably as a consequence of its larger particles size (132 nm). Differently, the 12sulf20 nanoparticle curve has reached the plateau rapidly due probably to both these factors: Its particle size (66 nm), which is almost a half of 30sulf20 nanoparticles size, which favors its permeation through rock pores, and its lower sulfonate group content which may have contributed to decrease its interaction with the sand.

The cumulative active substance recovered in the total effluent of those tests were 99.5%, 94.6% and 79.2% for 12sulf20, 30sulf20 nanoparticles and OADA surfactant solution, respectively. In other words, the losses in terms of mg of active/ mg of sand were 0.002 mg/g for 12sulf20, 0.025 mg/g for 30sulf20 and 0.096 mg/g for OADA. The adsorption of about 20% of OADA in the porous medium corroborates the concerns about surfactant adsorption and reinforced the necessity of nanocarrier employment. These results also proved that sulfonated polystyrene nanocarriers acted successfully, inhibiting the OADA adsorption onto sand particles surfaces. However, the increase of sulfonate content in the nanoparticles′ surface caused more adsorption onto the porous medium, but still, it was inferior to that observed for the only-surfactant solution. Due to that and of other properties explored in the previous sections, it was concluded that the 12sulf20 SPSNP forms the most effective system to percolate the porous medium in an unconsolidated sand column. Accordingly, these nanoparticles were further applied to an oil displacement test, which is discussed in the next section.

### 3.11. Oil Displacement Tests

**Figure 16:**
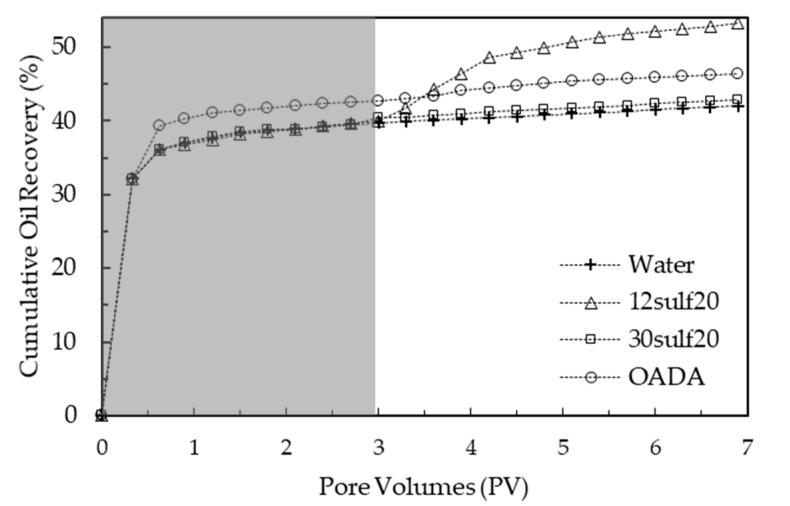
Oil recovery as a function of pore volume injected. The tertiary oil recovery began after the injection of 3 PVs of water.

Figure 16 shows the comparison between the oil recovery curves for the systems based on deionized water, OADA surfactant solution and nanoparticles suspensions. The shaded area is related to the secondary oil recovery, i.e., water flooding.

**Figure 17:**
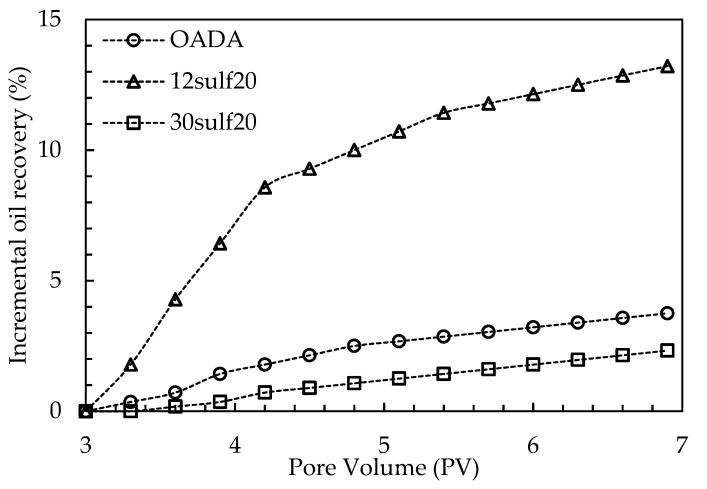
Incremental oil recovery as function of pore volumes after the secondary oil recovery.

The water flooding process produced a recovery in the range of 40–46% of the original oil in place in the same way as reported by other studies using sandstone as a porous medium [11,32,34]. In order to better discuss the system′s performance, Figure 17 displays the incremental oil recovery results from the chemical flooding stage, i.e., considering only the pore volumes injected after the secondary oil recovery.

It was observed that the OADA solution was not able to displace a satisfactory oil amount and this could be related to the fact that there is a great tendency of surfactant adsorption onto sand particles, shown by the transport test and discussed in the previous section, which causes losses during the flooding and therefore a worse oil displacement performance. 

The 30sulf20 nanoparticles presented a similar performance to the one presented by the OADA solution, promoting a discrete oil recovery. For this system, it is believed that the main factor could be from adsorption onto the porous medium, as suggested by the observed delay in its elution during the transport test, as discussed in the previous section.

In contrast, the suspensions containing the 12sulf20 nanoparticles enabled an incremental oil recovery of about 13% in relation to the water flooding oil recovery. This could be explained by the fact that the 12sulf20 SPSNP presented a high capacity to permeate through the porous medium, as shown in Figure 15, a significant IFT reduction (Figure 12) and a higher amount of OADA available (21.2 mg/g). In comparison with OADA solution, this surfactant nanocarrier conducted to a lower surfactant adsorption and consequently may have released a higher surfactant amount to the water/oil interface, reducing its IFT to lower values and enabling a higher displacement of residual oil. Therefore, the 12sulf20 SPSNP is the best surfactant delivery system developed in this work. In the same way, other studies in the literature observed that complex (dextrin-SDS) carrier systems were more efficient, achieving a similar incremental oil recovery in relation to the SDS flooding recovery in a sand porous media [32,34].

## 4. Conclusions

In this work, nanocarriers based on polystyrene containing sulfonate groups and the surfactant oleic acid diethanolamide (OADA) were developed. These particles presented controlled surfactant release when in contact with the oil phase, causing a great water/oil IFT decrease to very low values. Experimental investigations were performed to evaluate the influence of some polymerization reaction parameters, such as crosslinking agent, stabilizer and sulfonated comonomer amounts, on the main properties related to EOR application. Properties such as IFT, the retained surfactant amount in the nanocarrier and particle size were evaluated. Based on these results, the reaction conditions were optimized to an OADA concentration of twenty times CMC, 0.012 mol % of sodium 4-sulfonate-styrene, used as comonomer with styrene, and 3 (*v*/*v*) % of divinylbenzene, used as a crosslinking agent, obtaining the 12sulf20 SPSNP with 66 nm of particle size. These nanoparticles showed the best performance, since they reduced the water/oil IFT to 0.07 mN·m^−1^, improved stability in aqueous media, decreased the surfactant loss during the flooding on the sand surface and were responsible for a relevant gain in oil recovery when compared to free-surfactant solution. Overall, these partially sulfonated polystyrene nanoparticles have shown a potential to be used as surfactant nanocarriers for controlled release in enhanced oil recovery, since they were able to permeate through the unconsolidated porous sand medium, carrying a great amount of surfactant, increasing significantly the oil recovery by up to about 13% in relation to the water flooding oil recovery.

## Figures and Tables

**Table 1:** Formulations for nanoparticles polymerization reactions.

Sample	OADA ^1^ (g)	St ^2^ (mL)	St-S ^3^ (g)	St-S ^3^ (mol % of Total Monomer)
0sulf10	0.2888 (10CMC ^4^)	10.00	-	0
0sulf20	0.5779 (20CMC ^4^)	10.00	-	0
12sulf20	0.5779 (20CMC ^4^)	10.00	0.022	0.012
30sulf20	0.5779 (20CMC ^4^)	9.99	0.054	0.030

^1^ OADA: oleic acid diethanolamide. ^2^ St: styrene. ^3^ St-S: sodium 4-styrene-sulfonate. ^4^ CMC: critical micelle concentration.

**Table 2:** Particle size and Interfacial tension of PSNP obtained by polymerization with different OADA and DVB amounts.

Sample	OADA	DVB (*v*/*v*) %	Particle Size (nm)	PdI	IFT (mN·m^−1^)
01	10CMC	0.5	192	0.150	35.1
02	10CMC	1.5	176	0.282	17.9
03	10CMC	3.0	162	0.274	4.3
04	10CMC	4.5	128	0.226	12.1
05	10CMC	6.0	127	0.180	26.5
06	10CMC	9.0	138	0.136	29.7
07	20CMC	3.0	114	0.204	2.8

**Table 3:** Effect of sulfonate groups and OADA amount, used in the polymerization reaction, on the surfactant retention capacity of nanoparticles.

System	Particle Size (nm)	Retention%	*Q*_e_ (mg/g)
0sulf10	162	56.2	17.2
0sulf20	114	35.0	21.6
12sulf20	66	34.4	21.2
30sulf20	132	19.8	12.2
